# New insights into the poleward migration of tropical cyclones and its association with Hadley circulation

**DOI:** 10.1038/s41598-023-42323-7

**Published:** 2023-09-11

**Authors:** U. Anjana, Karanam Kishore Kumar

**Affiliations:** 1 Space Physics Laboratory, VSSC/ISRO, Trivandrum, India; 2 Kerala University, Trivandrum, India

**Keywords:** Atmospheric dynamics, Climate change, Climate-change impacts

## Abstract

Recent investigations have shown a robust signature of poleward migration of the tropical cyclone latitudes using observations and climate model simulations. Most of these studies invoked the role of the Hadley circulation (HC) expansion in the poleward shifting of tropical cyclones. However, none of these studies focused on the dissection of the zonally asymmetric HC into ascending and descending regions at regional scales, which holds the key in establishing the association between these two phenomena. Here, we are reporting the poleward migration of tropical cyclones and their association with ascending region boundaries of the HC at regional scales for the first time. The results emphatically show that the tropical cyclone latitudes as well as latitudes of maximum lifetime intensity vary in tandem with boundaries of the ascending region of the HC as compared to its descending region thus providing a vital clue on processes governing poleward migration of tropical cyclones.

## Introduction

Earth's temperature has been rising rapidly since the mid-twentieth century and the warming has increased from 0.08 to 0.18 °C per decade (more than double) since 1980^1^. The current reports show warming of more than 1 °C on average as compared to the pre-industrial era^2^. One of the important consequences of this warming is an increase in the intensity of extreme weather events. For example, it was shown that the impacts of typhoon Haiyan, which is the strongest storm ever recorded, were extreme because of climate change^3^. Recent investigations report an increase in intensity^4–7^ and a decline in the frequency of tropical cyclones (TC)^8–10^ along with their poleward migration^11–14^, which is attributed to anthropogenic-induced warming^15,16^. Another important consequence of the warming affecting the formation of TCs is the expansion of the Hadley circulation (HC)^17–19^. The HC expansion reported in the recent decades has a plethora of imprints^20^ such as poleward shifting of subtropical dry zones^21,22^, changing precipitation patterns^23–25^and poleward migration of TCs^26,27^.

In the beginning of the last decade, the phenomenon 'poleward migration of TC' was coined^14^. Initial reports showed that the latitude of lifetime maximum intensity (LMI) of TC migrated approximately 53 and 62 km per decade in the Northern and Southern hemisphere, respectively with a global migration rate of 115 km per decade according to global 'best-track' datasets^28^. A few studies showed that the inter-basin differences in the frequency of occurrence of TC affect the estimated trends in LMI latitudes^29^. It is noted that the trends in the northern hemisphere (NH) are contributed mostly by the differences in the frequency of TC rather than the migration of LMI. However, in the southern hemisphere (SH) observed trends are immensely contributed by the actual migration of TC^29^. Despite these disparities, a robust poleward shift of TCs over the majority of oceanic basins is noted in observations as well as climate model simulations. There has been a handful of investigations to identify the driving mechanisms for this phenomenon and the majority of them are associated with the HC expansion^26,30^. As both poleward migrations of TC and HC expansion share similar magnitudes (~ 0.5° to 0.8° latitude/decade), it was proposed that there might be an association between them. In a seminal study^30^ using multiple reanalysis datasets, it was reported that the HC edges and meridional distribution of TC vary concurrently. Further, it was reported that the weakening of HC, especially in the upper troposphere, plays a key role in the poleward migration of TCs^26^.

Recent studies on poleward migration of TCs linked this phenomenon to the HC expansion at regional as well as hemispheric scales^31–33^. However, the mechanism behind how HC expansion drives the poleward migration of TCs is yet to be ascertained. It is known that the ascending region of the HC plays a key role in triggering the tropical deep convection, which forms the basis for the formation of TCs. Any changes to the ascending region of the HC will have a profound impact on the TC formation as well as their propagation. Almost all the investigations on poleward migration of TCs focused on the total width of the HC rather than bisecting it into ascending and descending regions. There are no studies conducted so far on TC latitude distribution with respect to HC ascending region boundaries at regional scales. In this study, we provide new insights into the poleward migration of TC activity with respect to the ascending region of the HC at regional scales using 41 years of TC track observations and reanalysis datasets.

## Results

### Long-term evolution of TC latitudes

**Figure 1:**
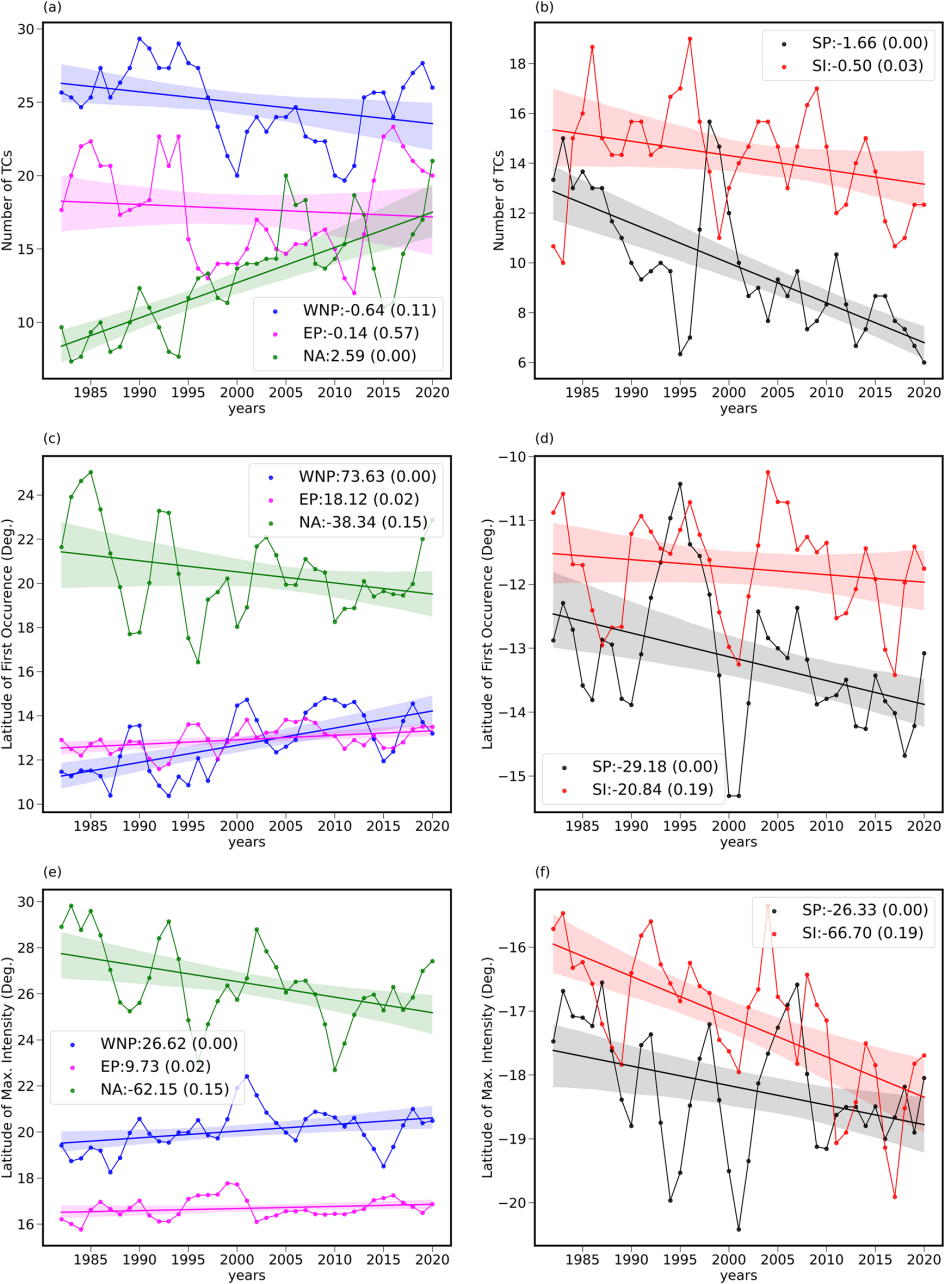
Long-term evolution of TC frequencies and formation latitudes. Left panel: time series of (**a**) annual mean number, (**c**) annual mean LFO and (**e**) annual mean LMI of TCs formed in the various oceanic basins in the Northern Hemisphere. Right panel: (**b**), (**d**) and (**f**) are the same as (**a**), (**c**) and (**e**), respectively for the oceanic basins in the Southern Hemisphere. A three-point running mean is applied for the time series. The observed trends along with p values in each oceanic basin are also provided in each graph. The observed trends are in number/decade for (**a**) and (**b**) and km/decade for rest of the graphs.

The TC track observations provided by the IBTrACS^28^ (International Best Track Archive for Climate Stewardship) and HC boundaries retrieved from ERA-5 reanalysis datasets^41^ are employed for the present investigation (refer to “Data and methodology” section). As reported by the earlier studies, there are robust signatures of poleward migration of TCs over most of the Oceanic basins. Here, we have carried out the trend analysis of the frequency of occurrence, the latitude of first occurrence (LFO), which is regarded as genesis latitude of TC and the LMI of TCs with extended datasets up to the year 2020 for each ocean basin as depicted in Fig. 1. Most of the earlier studies focused only on LMI citing the inaccuracies in determining the LFO^14,29^. The LMI is the most robust measure as this depends solely on the maximum intensity^14^. In this study, we attempt to use LFO without any subjective criterion along with LMI to verify its employability in future studies. As most of the earlier studies reported that ENSO has no significant effect on the estimated trends^26,30^, we have not removed the ENSO signal from the time series. However, there are evidences for Pacific Decadal Oscillation (PDO) and Interdecadal Pacific Oscillation (IPO) influencing the TCs formation in the West North Pacific (WNP) basin^34,35^ and North Atlantic Oscillation (NAO) in the North Atlantic (NA) Ocean. We will discuss the role of these oscillations in modulating the LFO and LMI in respective oceanic basins in the later part of this section. The time series over the North Indian Ocean is not included due to the inhomogeneities in the observations of TC tracks. From Fig. 1a,b, it is evident that the annual mean frequency of occurrence of TC is decreasing with time over all the oceanic basins except over the NA Ocean which shows an increasing trend. A rapid decline in the frequency is observed in the South Pacific (SP) at the rate of 1.66 per decade followed by 0.64 per decade in the WNP. However, the TCs in the NA are rapidly increasing at an unprecedented rate of 2.59 per decade. It is interesting to note that the LFO and LMI of TCs in the NA basin are relatively poleward (~ 18° to 24° N) when compared to all other oceanic basins (~ 10° to 14° in both the hemisphere) as observed in Fig. 1c,e. Further, it can be noted from the trends that the LFO is migrating poleward in all the oceanic basins except over the NA, which shows an equatorward migration. The migration rates along with p values representing the significance of the trends are depicted in the respective figures. The time series of LMI shown in Fig. 1e,f also show similar results as of LFO, however, with varying migration rates. It is interesting to note that the LFO of TCs in the WNP as well as Eastern Pacific (EP) is migrating towards poles relatively faster than their LMI counterpart. In SH, the poleward migration of both LFO and LMI show more or less similar migration rates in the SP whereas LMI shows a relatively faster poleward migration rate as compared to LFO in the South Indian (SI) Ocean in contrast to NH oceanic basins. In the case of the NA basin, the LMI equatorward migration is relatively faster than LFO.

**Figure 2:**
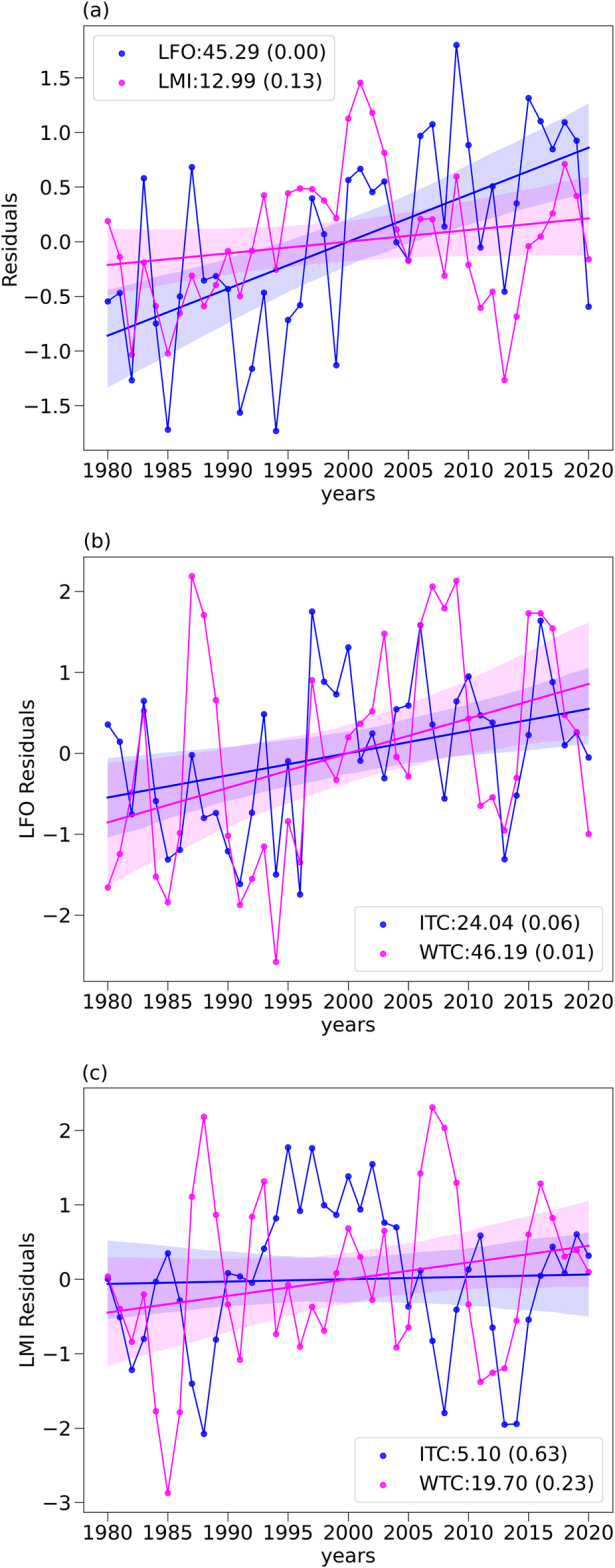
Estimation of residuals in LFO and LMI after removal of PDO/IPO signals and segregation of WTC and ITC. (**a**) Time series of residuals of LFO and LMI after the removal of IPO and PDO signals along with linear trends. (**b**) Time series of residuals of LFO for intense and weak tropical cyclones demarcated by a wind speed threshold of 65 knots. (**c**) Same as (**b**) but for LMI. The observed trends in km/decade along with p values are also provided in each graph.

As mentioned earlier, it is reported that PDO/IPO influences the TCs in the WNP basin. We have noted a significant negative correlation between PDO/IPO and LFO/LMI in line with the earlier reports^35^. It becomes essential to remove their effect while estimating the migration rates of TCs in the WNP. We have estimated the time series of residuals of LFO and LMI after removing the PDO/IPO signals from the original time series shown in Fig. 1c,e for WNP basin using multivariate linear regression technique. Figure 2a shows the time series of residuals of LFO and LMI along with the linear trends. It is interesting to note that there is significant reduction in the estimated migration rate after removing the PDO/IPO signals. The LFO migration rates are reduced to 45.29 from 73.63 km/decade (~ 38.5% reduction) and LMI rates are reduced to 12.99 from 26.62 km/decade (~ 51.2% reduction). The observed reductions in the migration rates after removing the PDO/IPO signal are comparable to those reported earlier^35^. The analysis emphasizes the role of natural variability in the observed migration rates of TCs. A similar analysis is carried out to remove the NAO signal from the LFO/LMI over the NA basin. However, an insignificant correlation is found between NAO index and LFO/LMI and there were no notable changes in estimated migration rates before and after removal of NAO signal as shown in the Supplementary Figure s1. Keeping this result in view, we have used the original time series of LFO/LMI over the NA basin for further analysis. The results from migration rate analysis are summarized in Table 1.

**Table 1:** Long-term trends in annual mean frequency, the latitude of first occurrence (LFO) and latitude of maximum intensity (LMI) observed in various oceanic basins.

Oceanic basins	Trends in annual mean frequency (/decade)	Trends in annual mean LFO (km/decade)	Trends in annual mean LMI (km/decade)
WNP*	− 0.64	73.63	26.62
EP	− 0.14	18.12	9.73
NA	2.59	− 38.34	− 62.15
SP	− 1.66	20.84	66.7
SI	− 0.50	29.18	26.33
*WNP (residuals)		45.29	12.99

Based on the maximum sustained winds, the TCs are classified as weak (< 65 knots) and intense tropical cyclones (≥ 65 knots)^36^ abbreviated as WTC and ITC, respectively. This classification is made to verify the relative contributions of WTC and ITC to the observed migration rates in the WNP basin. The analysis is carried out using both original time series as well as residuals of LFO/LMI. Figure 2b shows the residuals of LFO for WTC and ITC, which readily reveals that WTC contribute relatively more to the observed poleward migration rates of LFO as compared to ITC in the WNP. Similarly, Fig. 2c shows the residuals of LMI for WTC and ITC, which also shows dominance of WTC in the poleward migration. However, estimated trends in LMI of WTC and ITC are not significant at 95% as shown by the p values. The analysis of WTC and ITC for the original time series also carried out and the results are shown in Supplementary Figure S2a,b for LFO and LMI, respectively. From this figure, it is evident that the WTC migration rates are relatively larger than ITC for both LFO and LMI in the WNP basin, which confirms the robustness of the assertion that WTC dominates the poleward migration of TCs as reported earlier^36^. The potential reasons for the observed differences in the poleward migration of weak TC and intense TC in the WNP are discussed in an earlier study^36^.

### Bisecting HC into ascending and descending regions using Helmholtz decomposition

**Figure 3:**
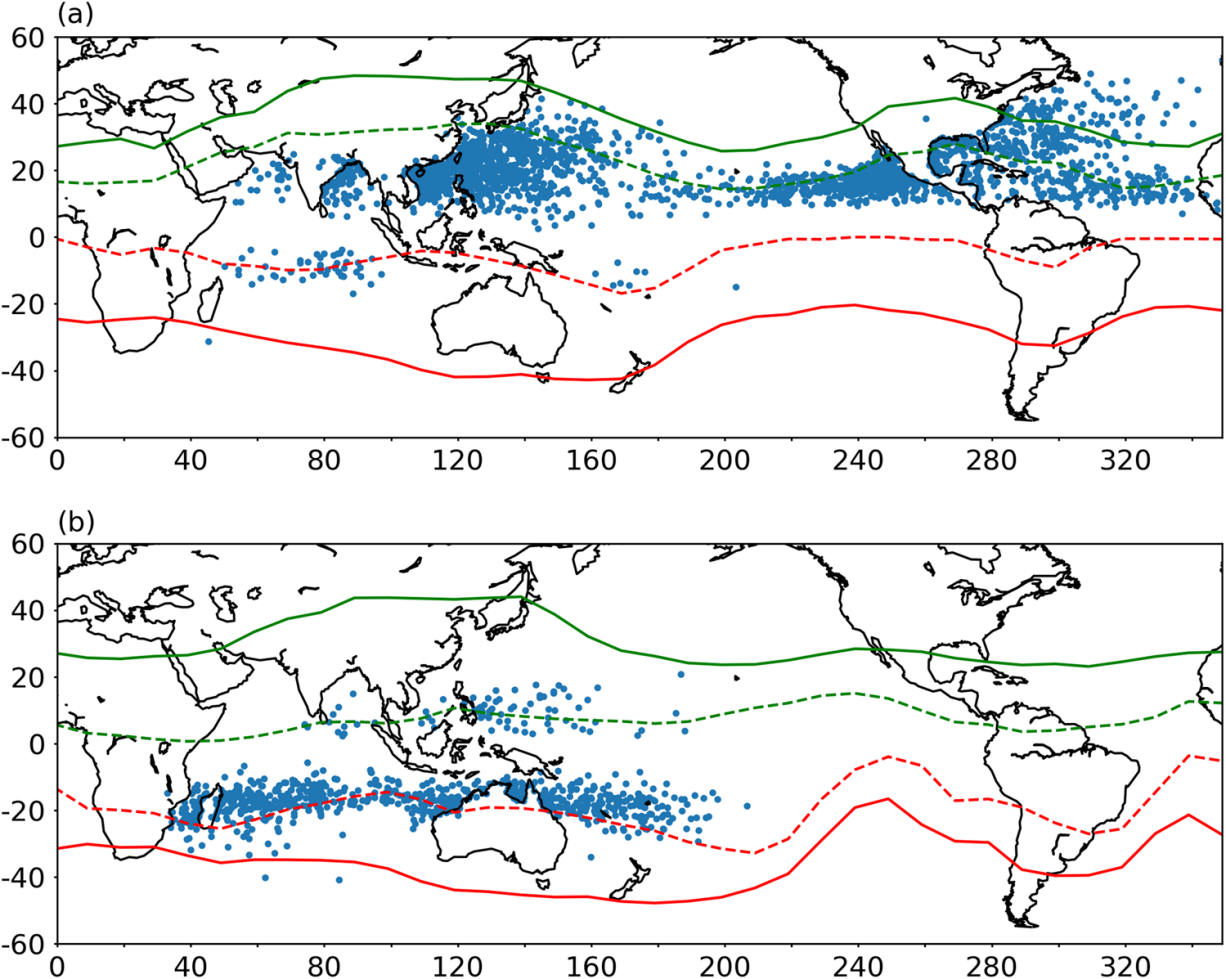
Distribution of LMI locations with respect to HC boundaries during peak TC season of NH and SH. The geographical locations of LMI during (**a**) JASO (July—August–September–October) for NH peak TC seasons and (**b**) JFM (January–February–March) for SH peak TC seasons. Red and blue solid lines represent the descending region boundaries and dashed lines represent the corresponding ascending region boundaries of the HC.

To provide further insights into the association between the poleward migration of TC and HC, we have used the zonally resolved HC boundaries at regional scales using Helmholtz's decomposition of winds (refer to “Methods” section). The seasonal climatology of HC ascending and descending region boundaries is shown in Supplementary Figure S3. The peak TC seasons differ from one ocean basin to other and the further analysis is carried out keeping these seasons in view. In the WNP, peak TC season is during July–August–September and October (JASO), in EP, it is during July–August–September (JAS) and in NA, it is during August–September and October (ASO). Thus TC peak season over the EP and NA is a subset of TC season over the WNP. Similarly, in the SH, the peak TC season over the SP and SI is during January–February March (JFM). Figure 3 shows the LMI of tropical cyclones formed during the past 41 years with mean HC ascending and descending region boundaries for peak TC seasons of NH (JASO) and SH (JFM). This kind of depiction provides new insights into the favourable locations for TC genesis with respect to HC boundaries. From this figure, it is evident that most of the TCs attain their maximum intensity around the boundaries of the HC ascending region in most of the ocean basins. However, there is a large spread of LMI over the WNP basin within the ascending region of the HC, which can be attributed to the large ascending region of the Walker cell residing over this region. Most of the LMI locations, however, are found to be around the ascending region of the HC. This observation provides a clue that the changes around the boundaries of the HC ascending region hold the key to poleward migration of the TCs. A few earlier studies associated the poleward shift of HC descending region boundaries and the TCs and found a moderate co-variability between the two^30^. There were no attempts in the past to examine the co-variability of ascending region boundaries with the TCs, which can be partly attributed to the lack of a zonally resolved metric for identifying the ascending region boundaries over the various oceanic basins. A similar depiction is made for the LFO of TCs and is shown in Figure S4, which shows that genesis locations are relatively equatorward as compared to LMI.

### Co-variability of HC boundaries and TCs

**Figure 4:**
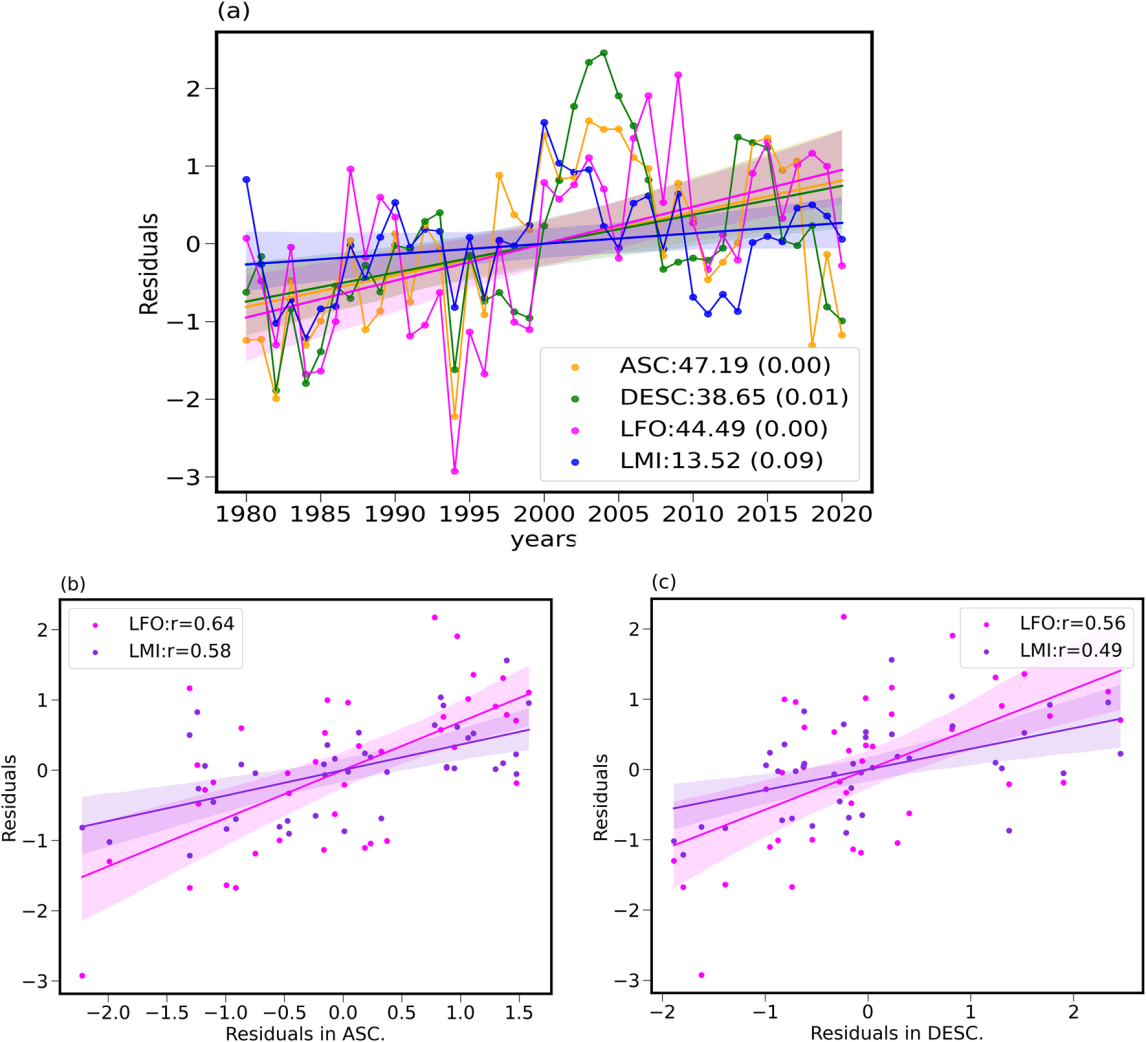
Co-variability of residuals of LFO and LMI with HC boundaries over the WNP after the removal of IPO and PDO signals. (**a**) Time series of residuals of LFO, LMI along with boundaries of ascending and descending regions of the HC over the WNP oceanic basin. All four time series are constructed after the removal of IPO and PDO. The observed trends in km/decade along with the p values are provided in the graph. The LFO and LMI residuals variations with respect to (**b**) HC ascending region boundary residuals and (**c**) HC descending region boundaries residuals. ASC and DESC in the legend of (**a**) corresponds to ascending and descending region boundaries of the HC. The solid lines in (**b**) and (**c**) represent the best-fit line and shading depicts the 95% confidence level intervals. The estimated correlation coefficients are provided in the respective graphs. Similar analysis without removal of IPO and PDO is shown in Supplementary Figure S5.

The peak TC seasons are identified over each oceanic basin as mentioned earlier and the co-variability between HC boundaries and TCs (LFO and LMI) are examined. Figure 4a shows the time series of LFO and LMI along with ascending and descending region boundaries of HC for the peak TC season i.e. JASO over the WNP. The analysis is carried out using time series with and without removing the PDO/IPO signals and Fig. 4 is for residuals and original time series is shown in Supplementary Figure S5. The PDO/IPO signal is also removed from HC boundaries in Fig. 4. All four parameters show a poleward shift with different migration rates. Again, the LFO/LMI as well as HC boundaries migration rates are estimated after removal of PDO/IPO signal. It is very interesting to note that the ascending region boundaries of the HC are migrating relatively faster than their descending region boundaries over the WNP. Figure 4b shows a scatter plot of LFO/LMI and the boundaries of ascending region of the HC over the WNP. It is very interesting to note a co-variability of both LFO and LMI with the ascending region boundaries with a correlation coefficient of 0.64 and 0.58, respectively. Figure 4c shows the correlation between HC descending region boundaries and LFO/LMI, which is found to be 0.56 and 0.49, respectively. This figure readily reveals that the TC parameters show relatively better co-variability with ascending region boundaries of the HC as compared to their descending region boundaries. The same conclusion can be drawn from Figure S5. This is one of the important outcomes of the present analysis. Similar analyses are extended to other oceanic basins and results are depicted in Supplementary Figures S6 (EP basin), S7 (NA basin), S8 (SP basin) and S9 (SI basin). The results are summarized in Table 2.

**Table 2:** Correlation coefficients of co-variability of ascending and descending region boundaries of HC with LFO and LMI over various oceanic basins.

Oceanic basins	Asc vs. LFO	Asc vs. LMI	Des vs. LFO	Des vs. LMI
WNP*	0.64	0.58	0.56	0.49
EP	− 0.23	0.41	− 0.46	− 0.02
NA	− 0.63	− 0.58	− 0.08	− 0.20
SP	0.42	− 0.02	0.36	0.05
SI	0.42	0.38	− 0.08	− 0.08

*Residuals.

It is evident from Table 2 that both LMI and LFO show a relatively higher correlation with ascending region boundaries over all the oceanic basins. However, as in the case of the number of TCs as well as long-term trends in LFO and LMI, the co-variability of ascending region boundaries with LFO and LMI shows a negative correlation over the NA. Though the correlation is negative over this basin, the magnitude is relatively high with the boundaries of ascending region (with LFO − 0.63 and with LMI − 0.58) as compared to their descending region (with LFO − 0.08 and with LMI − 0.20). The correlation of LMI is relatively higher with ascending regions (0.41) as compared to that with descending regions (− 0.02) over the EP. The correlation of LFO, however, shows a relatively better correlation with descending (− 0.46) as compared to ascending region (− 0.23). As noted in Fig. 1, there are no significant long-term trends (based on p values) in TC latitudes over the EP. In the SI basin, both LFO and LMI show relatively high co-variability with ascending region boundaries as compared to descending region and the same is the case over the SP. From Table 2, it can be concluded that both LFO and LMI show relatively higher co-variability with ascending region boundaries of HC as compared to their descending region counterparts, except over the EP. These observations thus provide an important assertion that LFO/LMI of TCs are relatively more sensitive to the movement of HC ascending region edges, which are not envisaged hitherto. This provides a clue on where to look for the processes responsible for the poleward migration of the TCs.

### Distribution of vertical shear of horizontal winds with respect to HC boundaries

**Figure 5:**
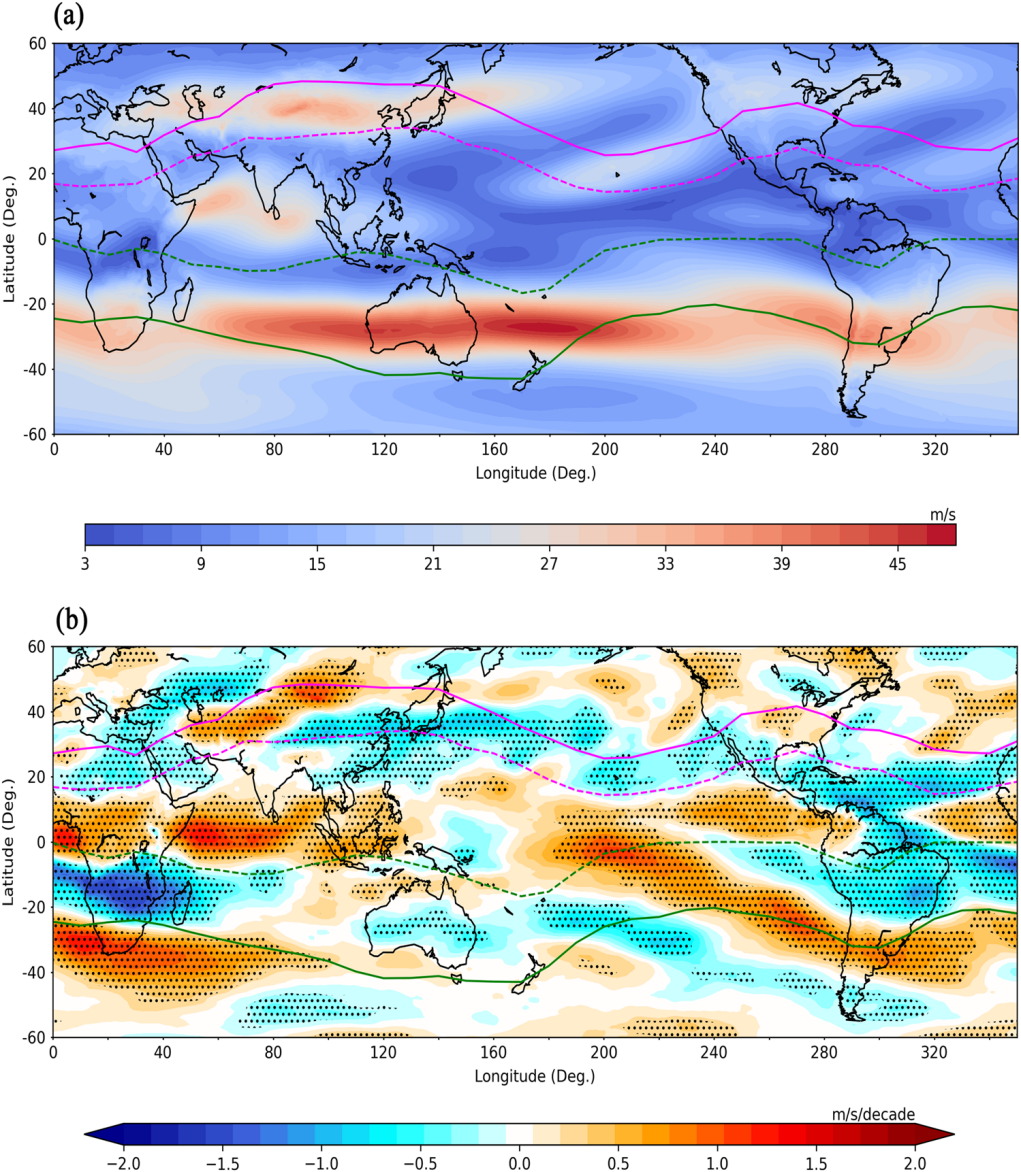
VWS distribution with respect to HC ascending and descending region boundaries during NH peak TC season (**a**) climatology of VWS and (**b**) trends in the VWS for JASO estimated using ERA5 reanalysis for the period 1980–2020. The magenta and green solid lines represent descending region boundaries and dashed lines represent the corresponding ascending region boundaries of the HC. The stippled areas in (**b**) represent the trends which are significant at 95% level.

The sea surface temperature, low-level vorticity, low-level convergence and vertical shear of horizontal winds are some of the most prominent parameters influencing the formation of TCs. Especially, changes in the HC dynamics can result in the redistribution of vertical shear of horizontal winds (VWS)^26^, which is one of the important factors in the formation of TCs. The regions with low VWS favour the formation of TCs as higher magnitude of VWS inhibits the vertical development of convective systems before cyclogenesis. To further investigate the VWS distribution with respect to the HC boundaries, its climatology for the WNP cyclonic season JASO is constructed and its long-term trends are estimated as shown in Fig. 5a,b, respectively. From Fig. 5a, it is evident that a region of low VWS, which is one of the favourable conditions for cyclogenesis, exists within the ascending region of the HC, especially near its edges. From Fig. 5b, it is very interesting to note that the majority of the WNP oceanic regions show an increasing trend in the VWS within the ascending region of the HC whereas a decreasing trend is found near the poleward edge of the ascending region. In general, there is a decreasing trend in the VWS in almost all the longitudinal sectors around the poleward edges of ascending region in the NH during this season. As the peak TC seasons of NA and EP basin are subset of WNP, separate analysis is not carried out for these two basins. In the NA, where the TC forms further north as compared to other oceanic basins, it can be noted that VWS shows a strong decreasing trend at the equatorward edge of the ascending region of the HC. This may be one of the potential reasons for the equatorward migration of TCs in this region as shown in Fig. 1c,e. This location coincides with the LFO and LMI of TCs in the NA as shown in Fig. 3a. The results thus show that one of the favourable conditions for the formation of TC is shifting poleward along with the ascending region of the HC potentially associated with changes in its intensity over most of the ocean basins except over the NA. A similar analysis is carried out for the SH cyclonic season (JFM) (refer to supplementary figure S10). This figure also shows similar results as those depicted in Fig. 5. The climatology of VWS shows relatively small values within the ascending region, especially at its edges. The long-term trends in VWS during this season show an increasing trend within the ascending region of the HC and decreasing trends at its poleward edges especially over the oceanic regions in the SH.

### Long-term trends in sea surface temperature

**Figure 6:**
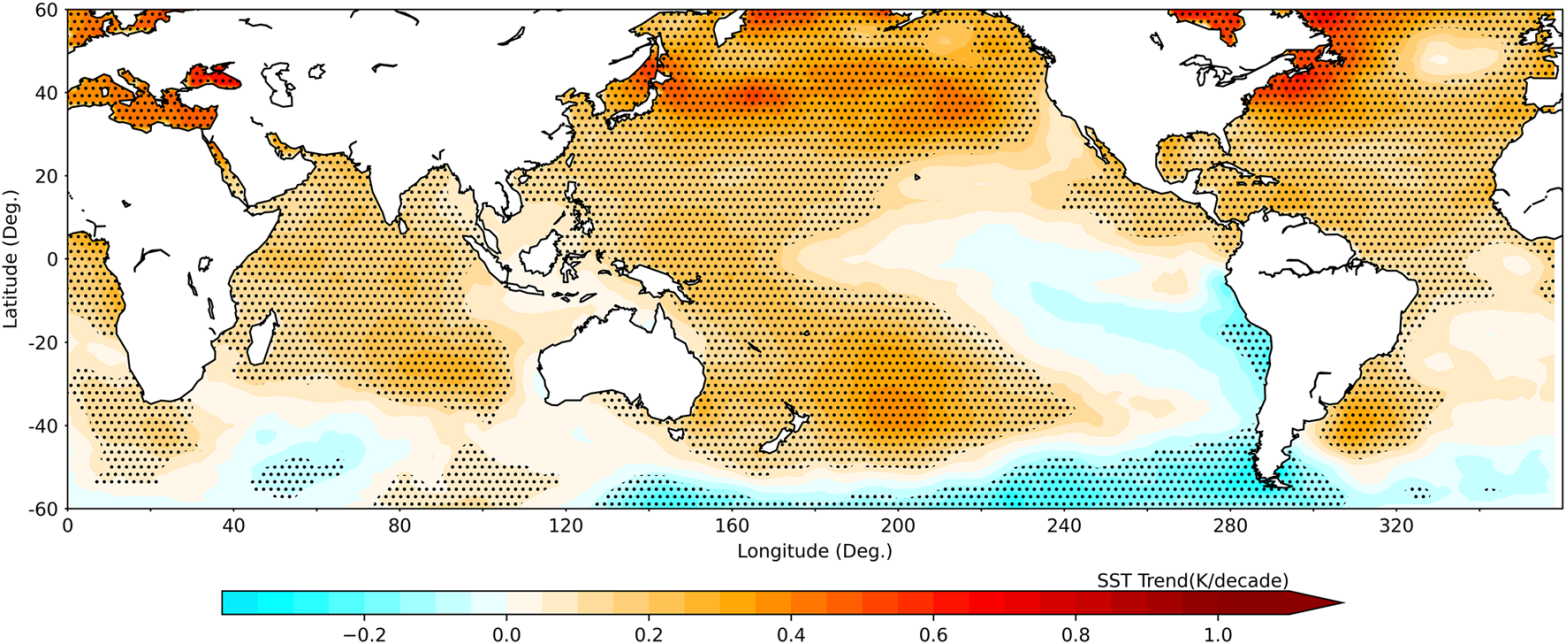
Long-term trends in the SST during NH peak TC season. Long-term trends in SST estimated using extended reconstructed sea surface temperature (ERSST) dataset in 2° × 2° grids for the period 1980–2020. The stippled areas represent the trends which are significant at 95% level.

The sea surface temperature (SST) and its variability in the tropical oceans play a key role in controlling the TCs formation as well as their propagation during their life-cycle^26,36^. Recent studies have reported a robust increasing trend in SST across the tropical as well as sub-tropical latitudes and attributed it to anthropogenic global warming. On the other hand, it is noted that the changes in meridional gradient in SST influence the HC dynamics^37^. Figure 6 shows the long-term trends in SST during the study period for JASO season. The trends are estimated in 2° × 2° grids and details are given in “Data and methodology” section. A significant increasing trend (~ 0.3 to 0.4 K/decade) can be noted over most of the latitudes with relatively large warming rates over the subtropical regions of WNP. The warming is significant at 95% level over most of the grids. An expanding HC has potential to modulate the SST response to the global warming^38^. The shifting of the descending region of the HC result in shifting of dry zones, which are void of clouds, towards north and thus resulting in relatively large warming rates over the subtropics as shown in Fig. 6, especially over the WNP^23,38–40^. The changes in latitudinal distribution of SST in turn alter the HC and associated dynamics including the large-scale dynamical conditions affecting the TC formation and intensification. The vertical air motions over the equatorial and low-latitudes, which form the ascending limb of the HC, depend on the SST and its meridional gradient. The SST play a key role in altering the ascending limb and hence the HC as discussed in a seminal study^37^. Thus, though the warmer SST associated with global warming in all the ocean basins in NH peak TC season is favorable for increasing the frequency of TC genesis, a decreasing trend is found as shown in Fig. 1 except for NA basin. This counter-intuitive observation is attributed to the changes in dynamical conditions associated with HC expansion. The SST trends for SH TC season JFM is shown in Supplementary Figure S11, which also reveals the warming of the oceans at the rate of ~ 0.3 to 0.4 K/decade. Earlier studies examined long-term evolution of maximum potential intensity (MPI), which is a key TC metric, and noted an increasing trend in most of the WNP basin with relatively higher rates in the northern sub-tropical latitudes. The spatial structure of MPI trends was very similar to that of SST^36^. The spatial pattern of SST changes is attributed to HC expansion^14^. However, it is to be noted that the seasonal variation of MPI in the WNP is also contributed by upper-level outflow temperature apart from the SST. The SST analysis thus shows an overall warming of the sea surface consistent with global warming and meridional gradient in the warming rates having a potential to modulate the large-scale circulation.

## Discussion

The time series of the number of TCs forming in the various ocean basins show a robust decreasing trend in almost all the basins except in the NA, where it shows an increasing trend. The large decline in the number of TCs in a year is noted in the SP (~ 6.8 in 4 decades.) followed by WNP (~ 2.62 in 4 decades). It is also noted that both LFO and LMI show poleward migration in the majority of the oceanic basins except in the NA, where it shows an equatorward migration. The PDO/IPO, which has known influence on the TCs forming in the WNP basin, has shown a significant negative correlation with LFO/LMI. It is noted that the observed poleward migration rates of LFO and LMI are reduced by 38.5 and 51.2%, respectively. However, no significant changes are observed in the migration rates of LFO and LMI after and before removing the NAO index signal in the NA basin. Further, the analysis carried out separately for weak and intense TCs has revealed that the WTC contributes relatively more to the poleward migration rates of TCs. Though a few earlier works studied the poleward migration of TCs with HC expansion, none of them focused on the dissection of HC further into ascending and descending region boundaries. The present analysis focused on the zonally resolved ascending and descending region boundaries using Helmholtz decomposition of horizontal winds and investigated the poleward migration of TCs with respect to these boundaries. The LMI distribution showed that most of the TCs occur around the boundaries of ascending region of the HC in the respective hemisphere in most of the oceanic basins. This observation provided a clue that the ascending region boundaries of the HC may be holding the key in the observed poleward migration of the TCs. Further investigations showed that the ascending and descending region boundaries of the HC are moving poleward at different rates over most of the oceanic basins and the TCs show significantly higher co-variability with the ascending region boundaries. Both residuals as well as original time series of LFO and LMI have shown significant co-variability with HC boundaries in the WNP. This perspective is emerged for the first time, which provides new insights into this phenomenon.

The changes in HC dynamics in the recent past may have a potential impact on the VWS distribution, which is one of the important parameters associated with TC formation. The climatology of VWS has shown a low wind shear regime around the poleward edges of ascending region, which is a conducive condition for tropical cyclogenesis. The long-term trends in VWS show enhanced magnitude mostly within the ascending region and reduced magnitude around its poleward edges over most of the ocean basins, in general. This is one of the important aspects of both poleward migrations as well as the reduced number of cyclones in a given ocean basin except in the NA. It is known that the conditions within the ascending region of the HC are relatively more conducive for cyclogenesis in terms of higher SST compared to its poleward latitudes. The high SST triggers the mesoscale convection over the oceanic regions and depending on the VWS, low-level vorticity and Coriolis parameter, these convective systems organise themselves to initiate the cyclogenesis. As noted in the present study, the TCs are shifting poleward, which can be attributed to the strengthening of VWS within the ascending region of the HC and weakening at its edges associated with the changes in the intensity of the HC during recent times. However, as favourable VWS conditions are shifting poleward where SST is relatively low as compared to its equatorward latitudes, the number of TCs is showing a decreasing trend in most of the oceanic basins. The number of TCs seems to be a trade-off between reduced VWS (favourable condition) and reduced SST (unfavourable condition). The long-term trends in SST have shown an overall warming of the oceans with relatively large warming rates at the subtropical zones over the WNP basin leading to meridional gradients in the SST, which in turn can affect the HC dynamics. The SST is a key factor for the TC genesis and the latitudinal gradient in SST is key for the HC dynamics.

The observations over the NA show an equatorward migration of TCs and an increase in the number of TCs. Interestingly, over this oceanic basin, the VWS is decreasing around the equatorward edges of the ascending region of the HC whereas it is increasing over other ocean basins, especially over the Pacific during the JASO season as shown in Fig. 5b. As favourable VWS conditions persist at the equatorward of ascending region boundary of HC where SST is relatively high, the TCs are increasing in number in the NA. However, the observations over the NA basin show equatorward migration of TCs and poleward migration of HC with significant negative correlation. The potential mechanism behind these observations over the NA basin needs further investigations. The HC expansion is driven by global warming due to increased greenhouse gas emissions^33^ and this phenomenon in turn affects the regional climate by modulating the various geophysical processes in and around the ascending and descending region boundaries of the HC. The present results can serve as key inputs for climate modellers as they shed light on the pathways through which HC expansion is affecting TCs. Most of the climate models are forecasting that the HC expansion will continue till the end of this century^33^, which may result in further poleward migration of TCs. This may further alter the established precipitation patterns associated with TCs and expose the biota living in the subtropical regions to the wrath of these extreme weather events.

## Methods

### Data

The present study used the IBTrACS^28^ (International Best Track Archive for Climate Stewardship) dataset for the analysis of TC climatology and trends and the state-of-the-art ERA-5 reanalysis dataset^41^ for characterising the HC at regional scales. The parameters of TCs viz., the latitude of first occurrence(LFO) and latitude of maximum intensity (LMI) are extracted from the IBTrACS whereas the monthly mean zonal and meridional winds of ERA-5 reanalysis are used for the computation of Meridional Mass Stream function and thereby, the HC boundaries during 1980–2020.

### Climatology and trend analysis of tropical cyclone latitudes

IBTrACS provides the 'best track' measurements of a TC, which includes the best estimate of the TC position, maximum sustained wind speed, and central pressure on a 6-h basis^28^. The parameters of TC considered for analysis are annual averaged LFO, LMI and frequency of occurrence. All TCs with maximum sustained wind speeds greater than 33 knots are considered for the analysis. The trend analysis including a significant test is carried out using Mann–Kendall Test using the Pymann–Kendall module in Python.

### Zonally resolved Hadley cell boundaries

Most of the earlier studies have employed zonal mean meridional mass stream function (MSF)^42^ to describe the HC as these estimations require mass conservation. The present study retrieved zonally resolved HC boundaries from ERA-5 reanalysis^41^ by employing Helmholtz decomposition of horizontal winds into divergent and non-divergent components using windspharm module^43^. Subsequently, the meridional component of divergent winds is used to compute the mass stream function using the following equation,

$$\psi \left(\theta ,\phi ,p\right)=\frac{2\pi a \mathrm{cos}\theta }{g}{\int }_{0}^{p}v\left(\theta ,\phi ,p\right) dp$$

The mean mass stream function between 400 and 800 hPa levels is used for retrieving the boundaries of HC (both ascending and descending regions) as demonstrated in Supplementary Figure S1. The latitude where MSF is maximum (minimum) in NH (SH) is identified as the edge of the ascending region shown by dashed lines in the figure. Thereafter, the first occurrence of zero poleward from these maxima/minima is identified as the edges of descending regions of the HC. In cases, where there is no zero crossing MSF, the latitude of local minima (minimum between ascending region edge and 40° latitudes) is identified as the HC edge. These HC boundaries are used to investigate their co-variability with TCs.

### Vertical wind shear

Vertical wind shear (VWS) of horizontal winds is estimated from zonal and meridional winds at 200 and 850 hPa using the following equation,

$$VWS=\sqrt{{\left({u}_{200}-{u}_{850}\right)}^{2}+{\left({v}_{200}-{v}_{850}\right)}^{2}}$$

### Supplementary Information

Supplementary Information.

## Data Availability

The data for TC parameters are taken from IBTrACS dataset (https://doi.org/10.25921/82ty-9e16). Monthly mean horizontal winds for evaluating HC boundaries as well as VWS is taken from ERA-5 reanalysis data (available at https://cds.climate.copernicus.eu/). The indices for natural variabilities such as IPO/PDO and NAO are taken from the corresponding NOAA websites (https://psl.noaa.gov/data/) and (https://www.ncei.noaa.gov/access/), respectively^44^. NOAA Extended Reconstructed Sea Surface Temperature (ERSST), Version 5 dataset was used for monthly mean gridded Sea Surface Temperature analysis and is available at (https://www1.ncdc.noaa.gov/)^45^.
