# Supplementary material for: New insights into the poleward migration of tropical cyclones and its association with Hadley circulation
Source: Sci Rep. 2023 Sep 11;13:15009. doi: 10.1038/s41598-023-42323-7 (PMC10495354; doi:10.1038/s41598-023-42323-7)
Supplement: Supplementary file 1 — Supplementary Information. [file 41598_2023_42323_MOESM1_ESM.pdf]

Supplementary Material

New insights into the Poleward Migration of Tropical Cyclones and its association with Hadley Circulation

U Anjana [1, 2] and Karanam Kishore Kumar [1]
[1]Space Physics Laboratory, VSSC/ISRO, Trivandrum
[2]Kerala University, Trivandrum

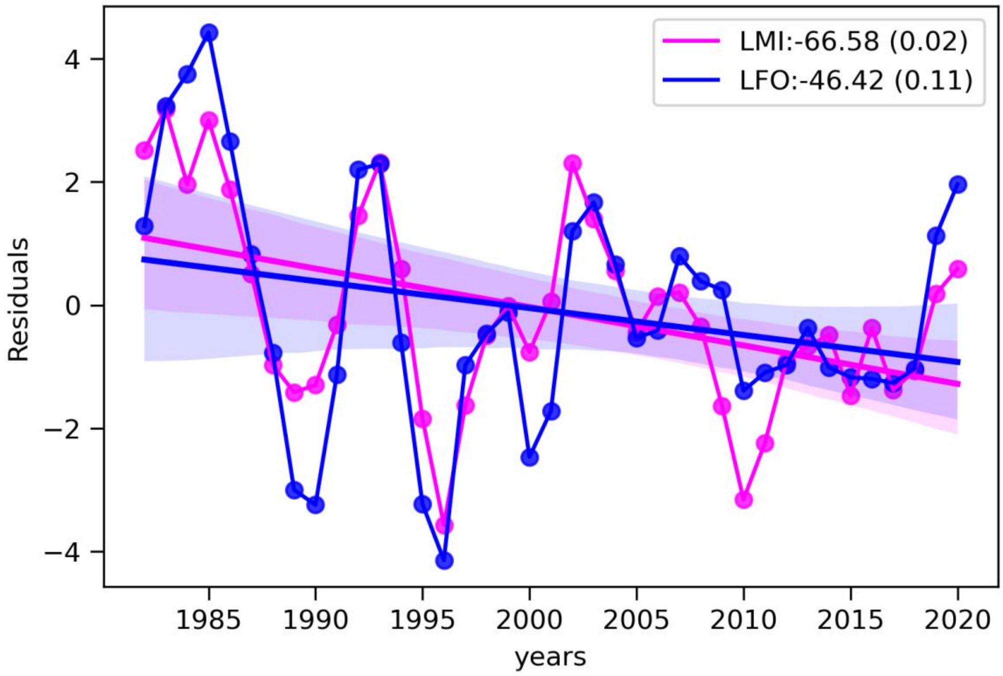

Figure S1: Verification of influence of the NAO on LFO and LMI in the NA basin. Time series of the residuals of LFO and LMI after the removal of NAO in the NA basin. The observed trends in km/decade for both LFO and LMI residuals along with p-values are provided in the graph

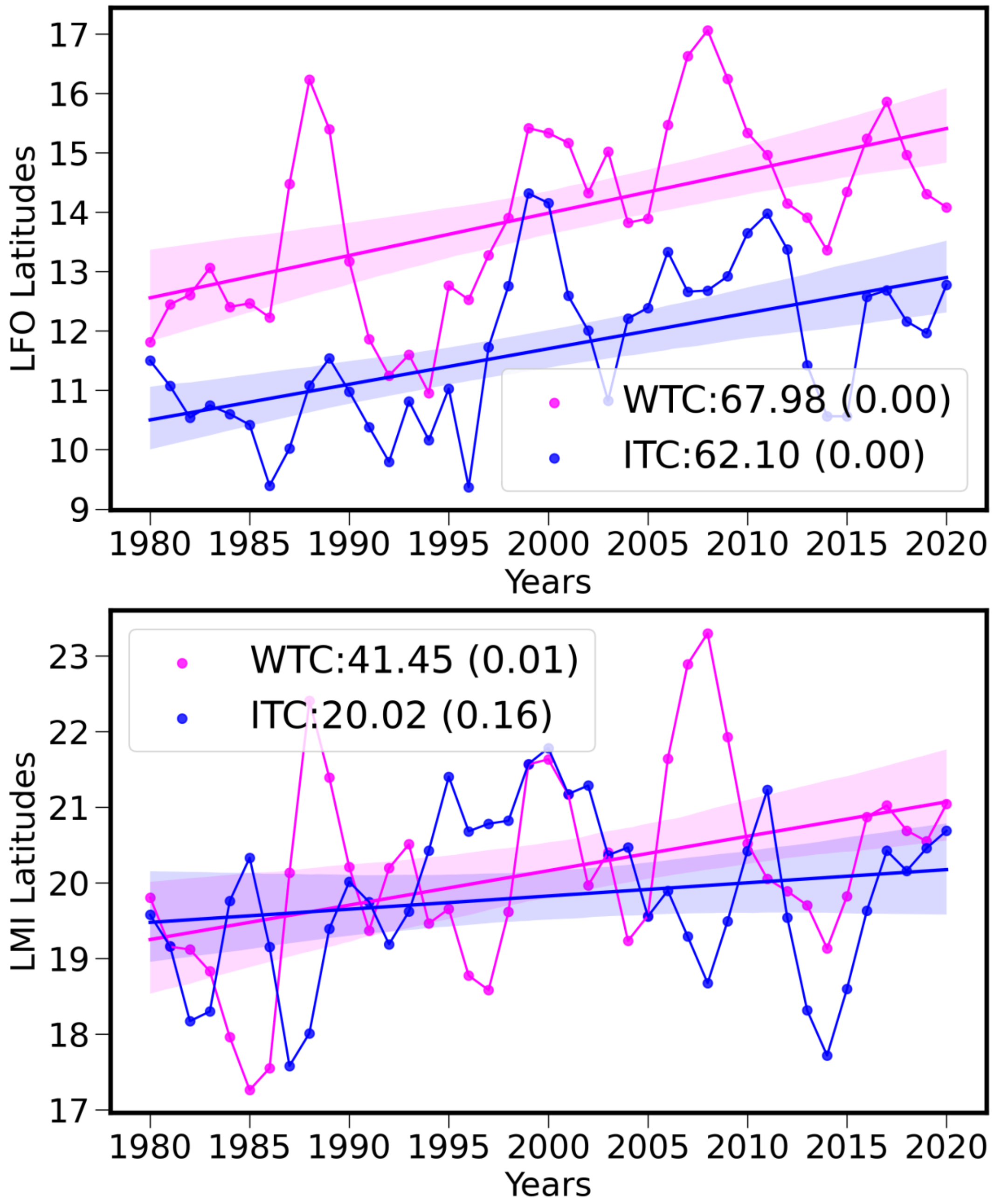

Figure S2: Analysis of WTC and ITC migration rates. The annual mean time series of (a) LFO and (b) LMI for WTC and ITC. The WTC and ITC are demarcated by a threshold wind speed of 65 knots. The observed trends in km/decade for both LFO and LMI residuals along with p-values are provided in the graph

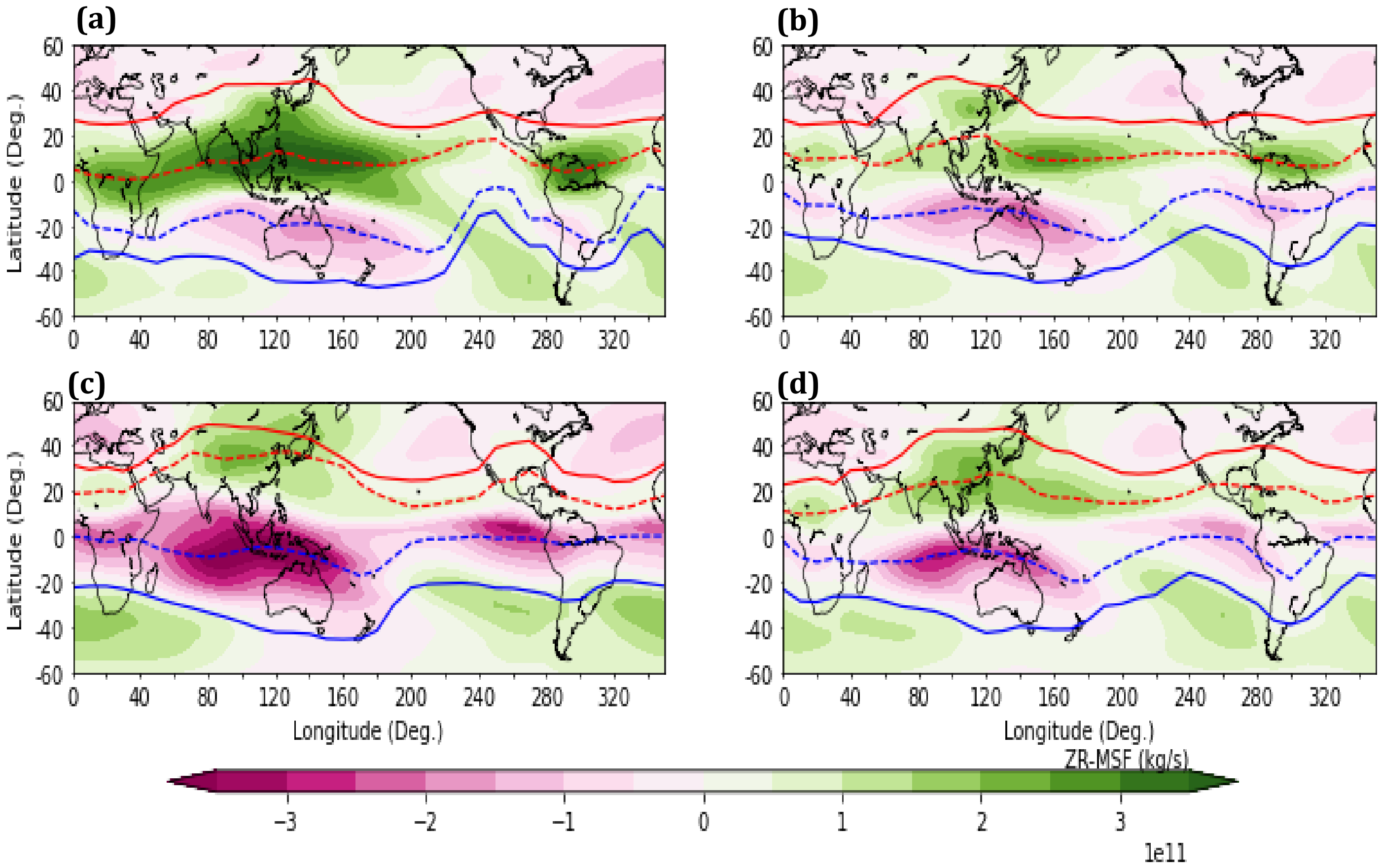

Figure S3: Climatology of HC ascending and descending region boundaries. The MSF estimated from meridional component of divergent winds and averaged in the 400-800 hPa levels during (a) December-January February, (b) March-April-May, (c) June-July-August and (d) September-October-November. The dashed (solid) lines represent the ascending (descending) region boundaries of the HC in NH (red) and SH (blue).

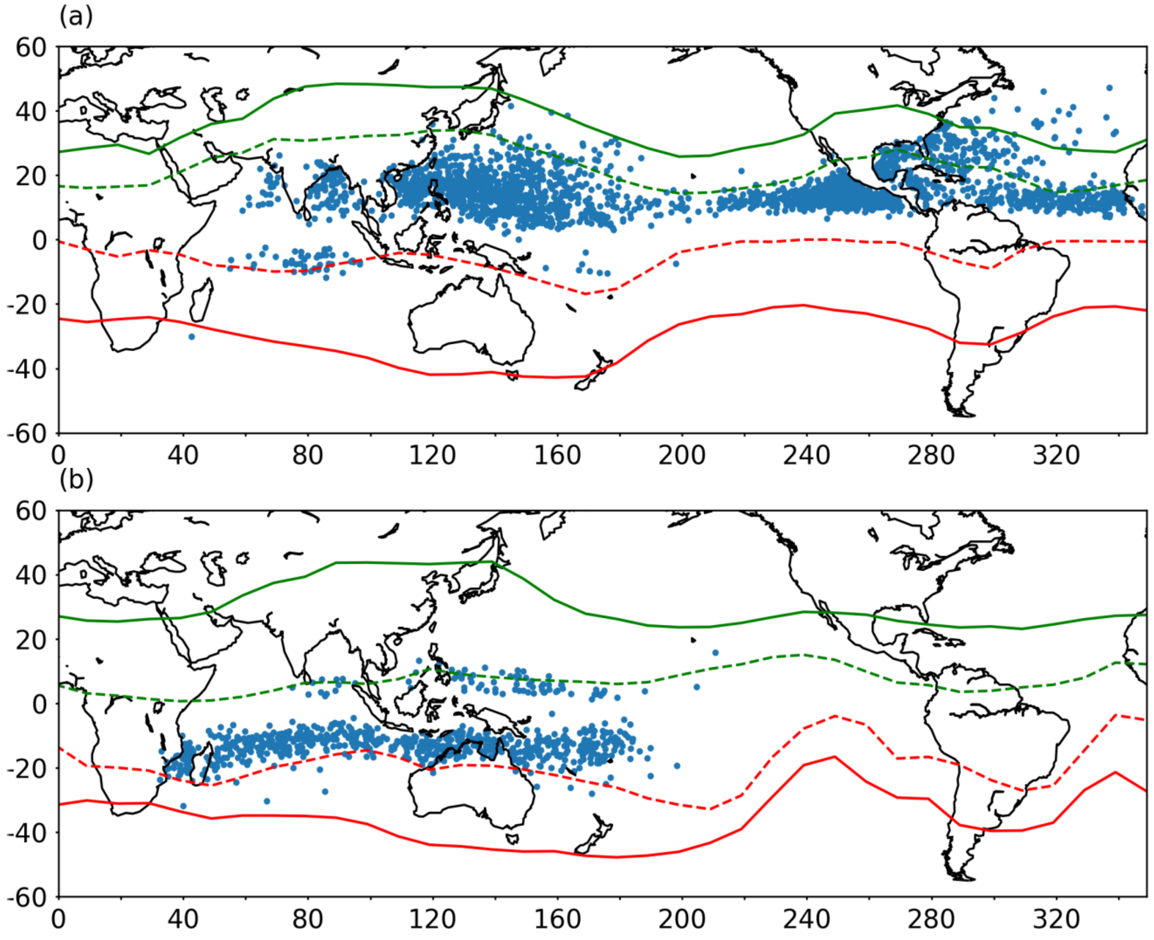

Figure S4: Same as Figure 3 but for LFO

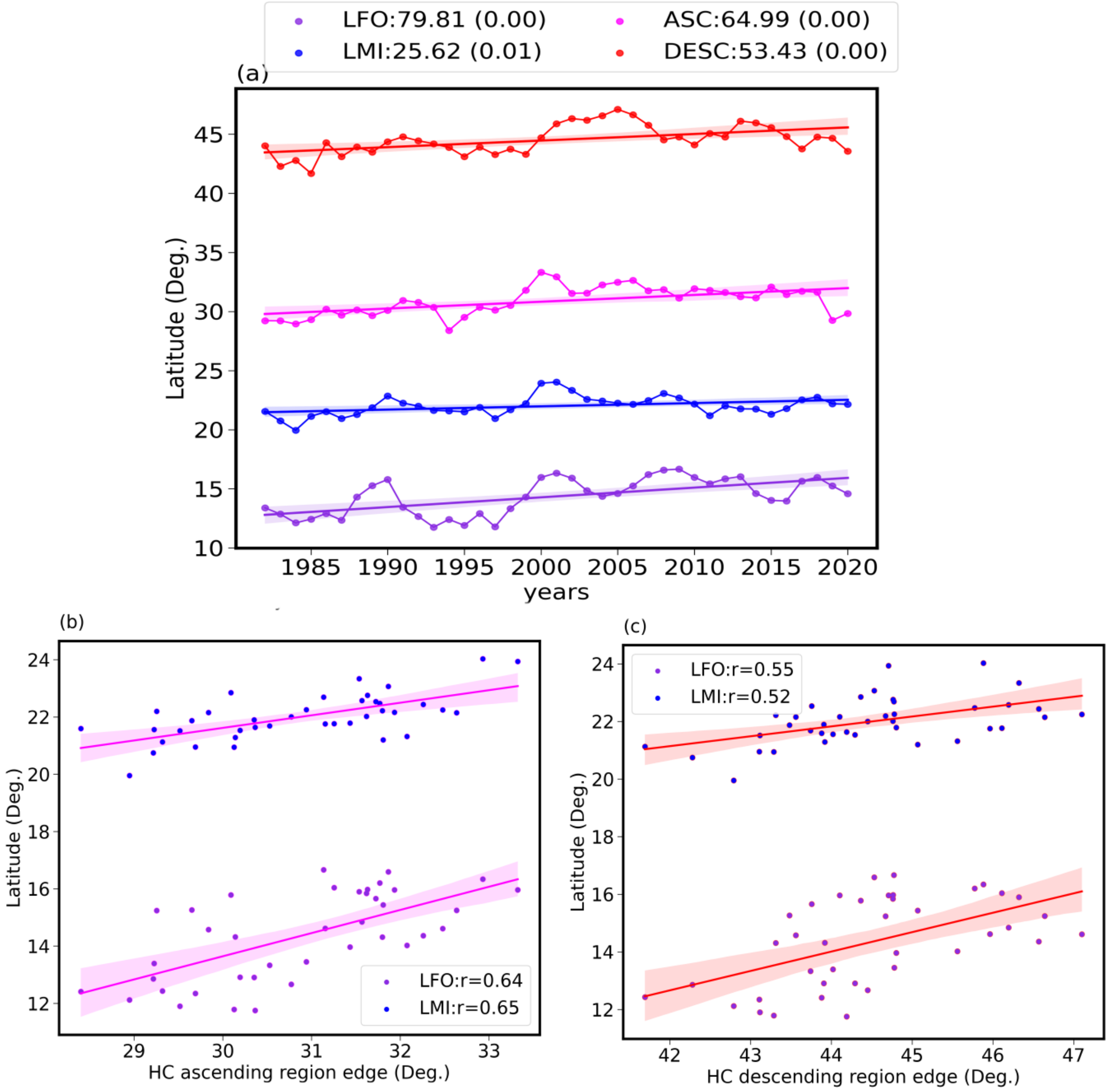

Figure S5: Co-variability of LFO and LMI with HC boundaries over the WNP without removing the PDO/IPO signals (a) Time series of LFO, LMI along with boundaries of ascending and descending region boundaries of the HC over the WNP oceanic basin. The observed trends in km/decade along the p values are provided in the graph. The LFO and LMI correlation with (b) HC ascending region boundaries and (c) HC descending region boundaries. ASC and DESC in the legend of (a) corresponds to ascending and descending region boundaries of the HC. The solid lines in (b) and (c) represent the best-fit line and shading depicts the 95% confidence level intervals. The estimated correlation coefficients are provided in the respective graphs.

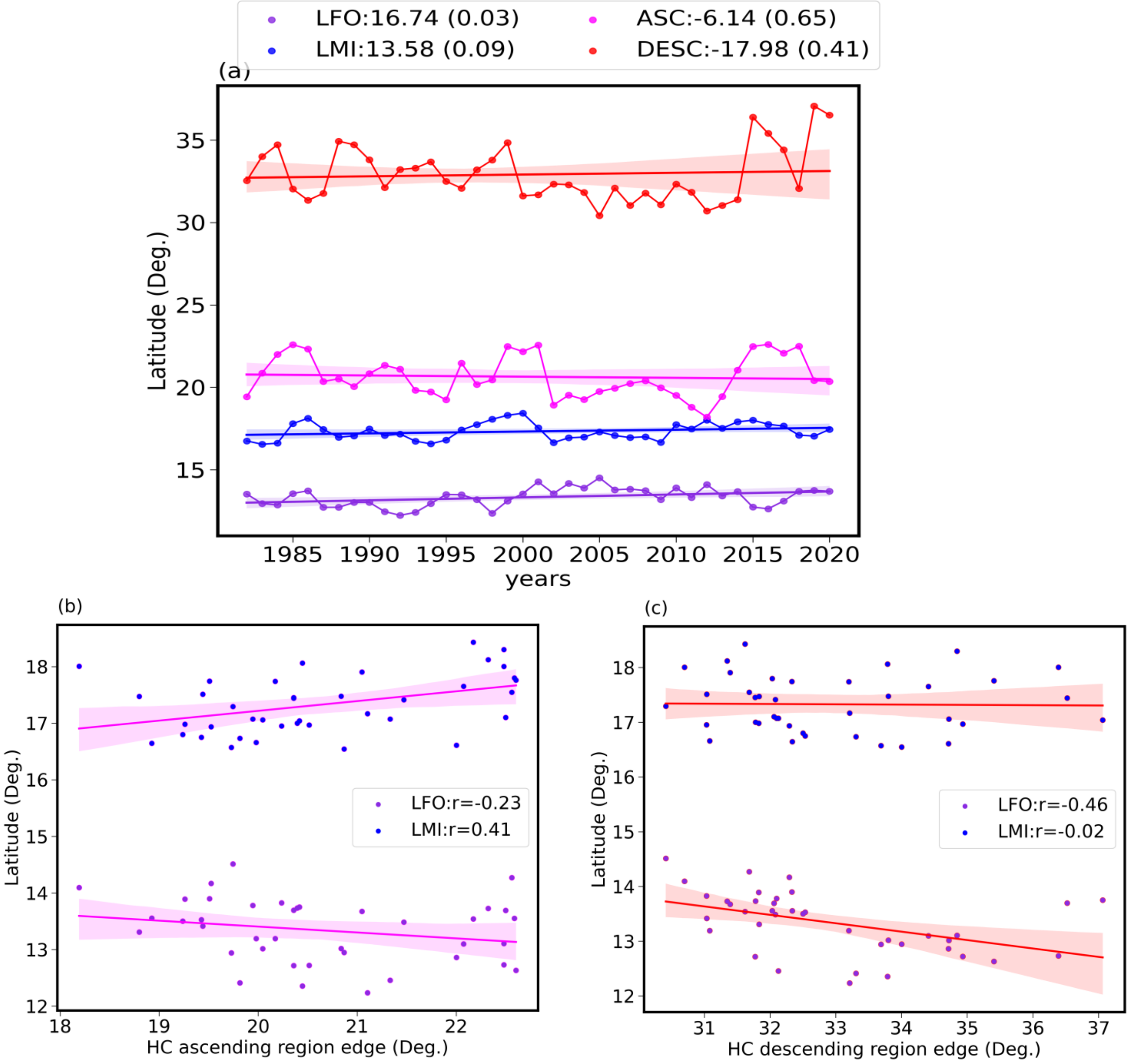

Figure S6: Same as Figure S5 but for the EP

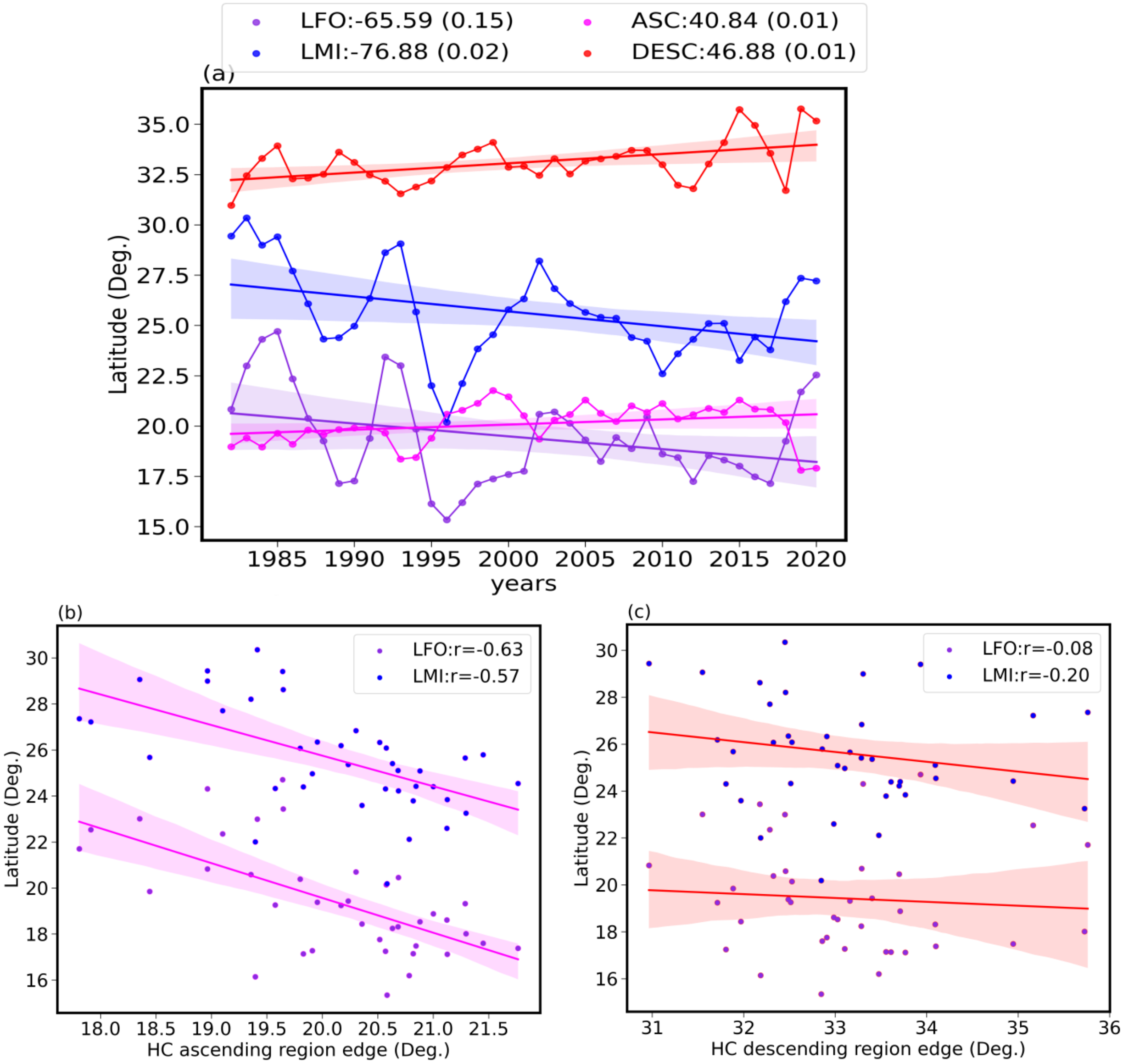

Figure S7: Same as Figure S5 but for the NA

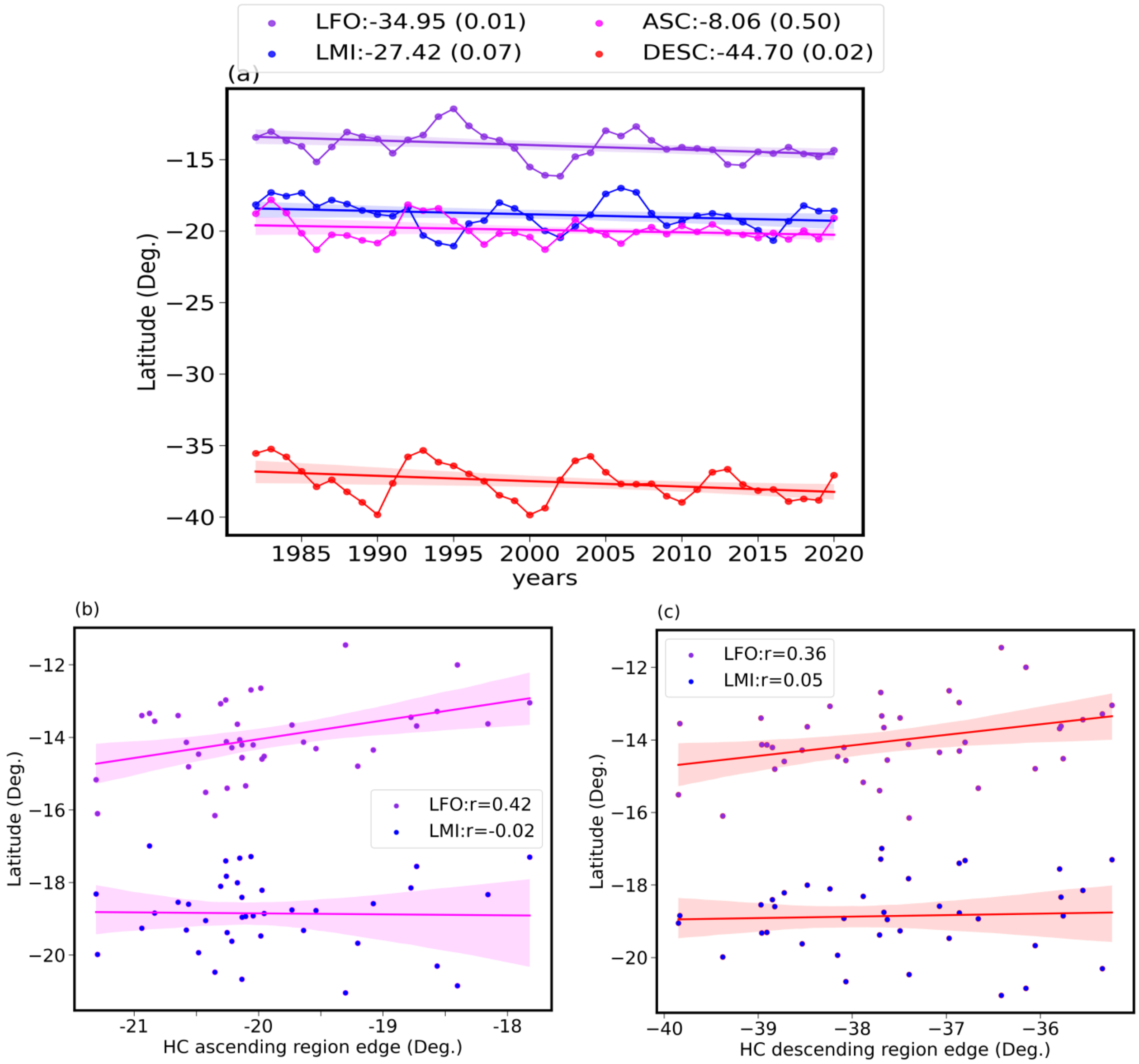

Figure S8: Same as Figure S5 but for SP

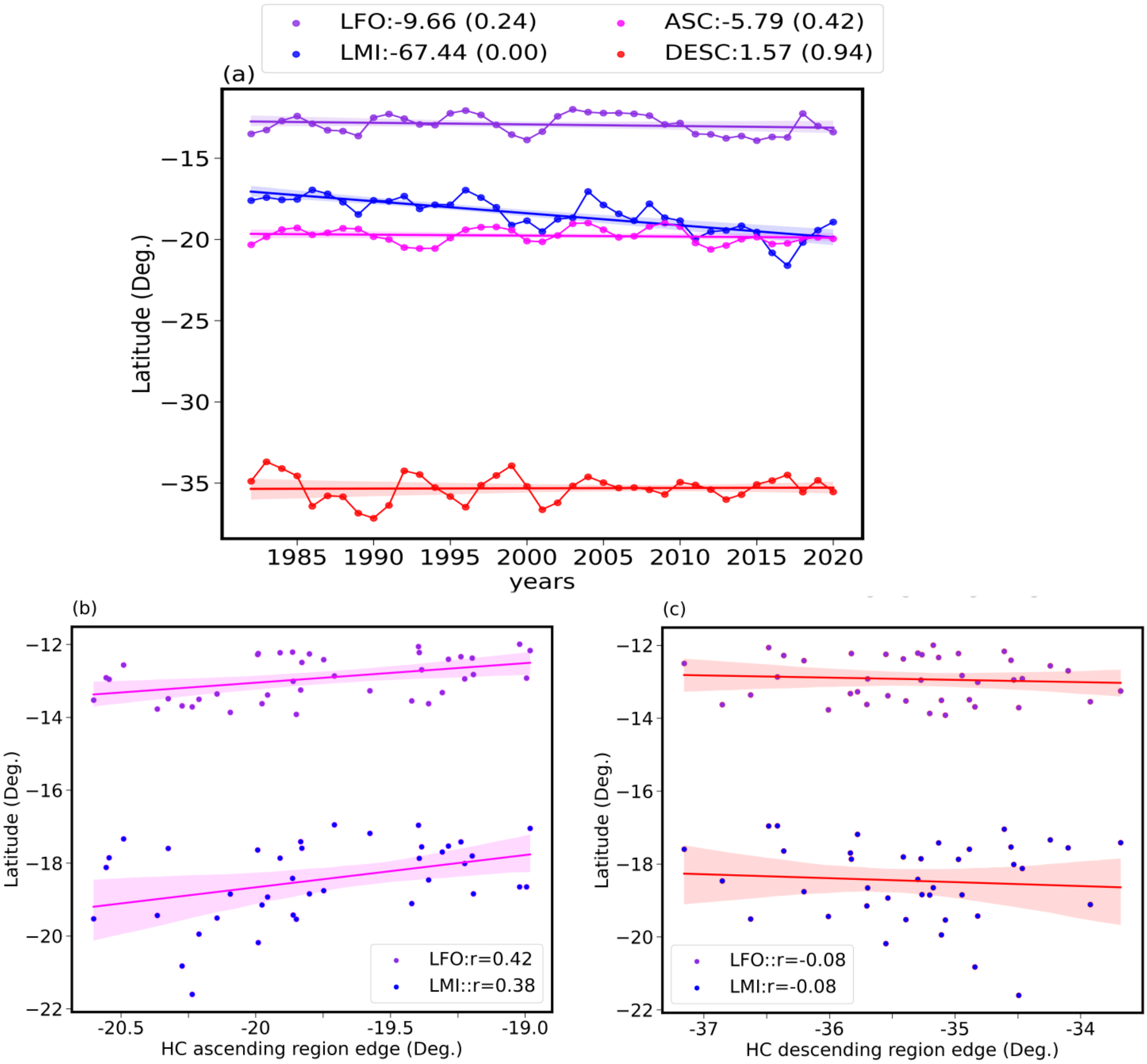

Figure S9: Same as Figure S5 but for SI

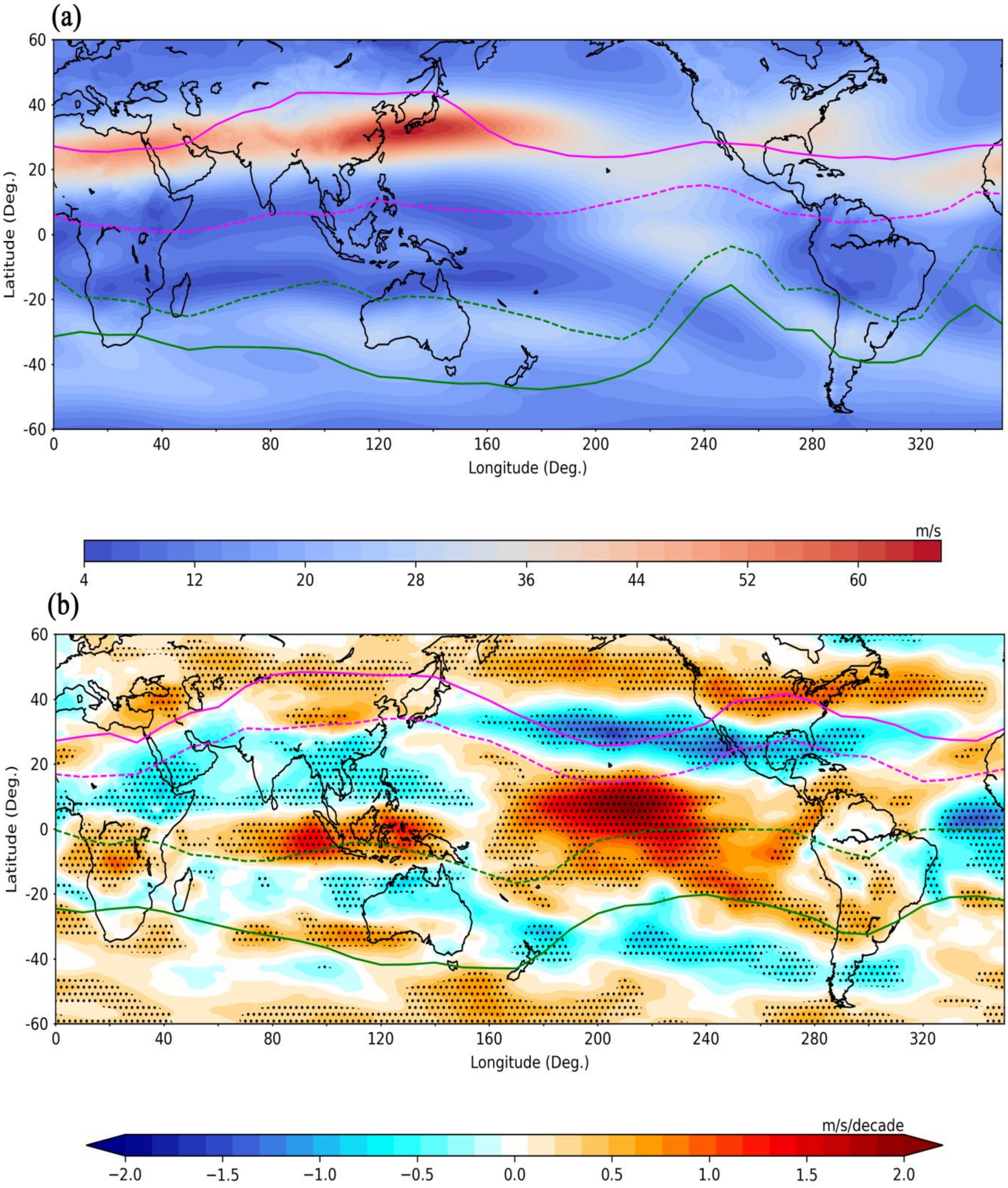

Figure S10: Same as Figure 5 but for JFM

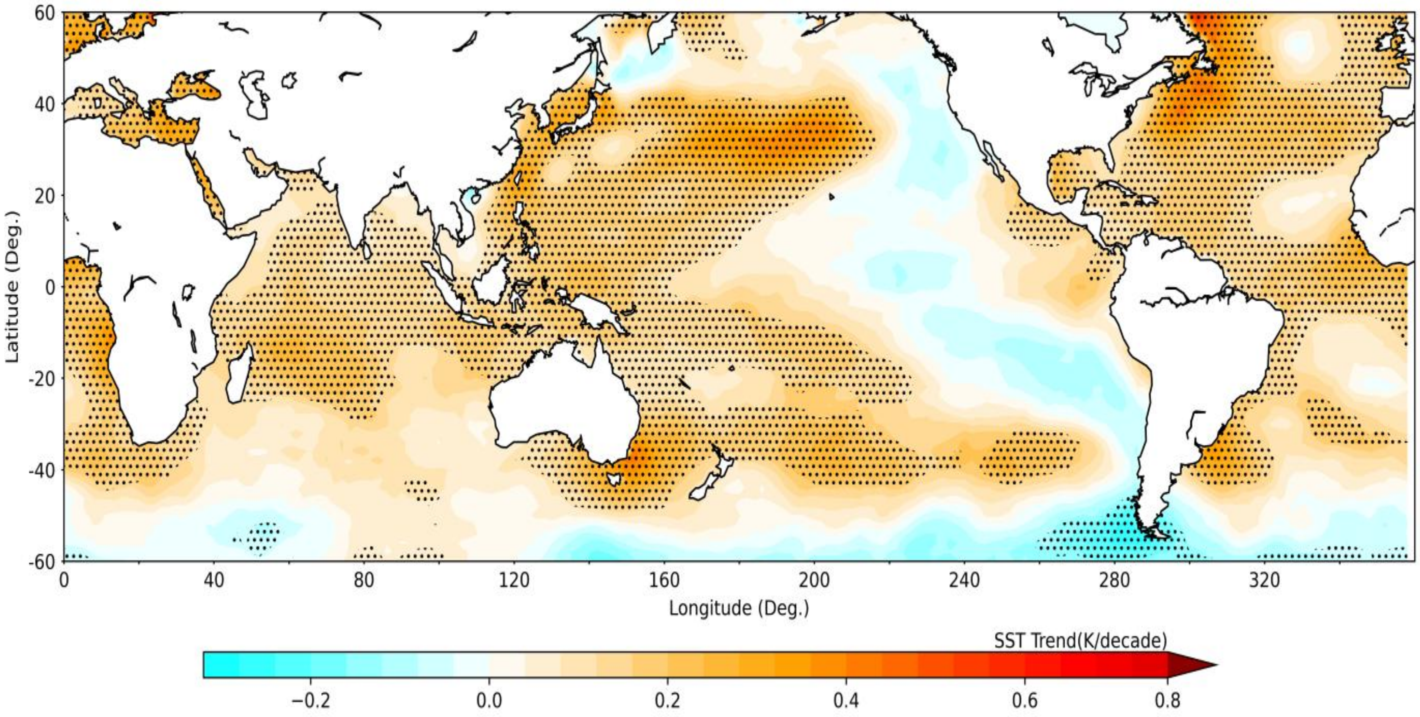

Figure S11: Same as Figure 6 but for JFM
